# Animal Abuse and Neglect in Companion-Animal Practice: The Role of Training, Legislation, and Veterinarian–Client Relationships in Romania

**DOI:** 10.3390/vetsci13070696

**Published:** 2026-07-17

**Authors:** Adela Ioana Mustăţea, Violeta Ștefania Rotărescu, Adrian Gorbănescu, Emilia Ciobotaru-Pîrvu

**Affiliations:** 1 Faculty of Veterinary Medicine, University of Agronomic Sciences and Veterinary Medicine of Bucharest, 105 Splaiul Independenței, District 5, 050097 Bucharest, Romania; emilia.ciobotaru-pirvu@fmvb.usamv.ro; 2 Faculty of Psychology and Education Sciences, University of Bucharest, 90 Şoseaua Panduri, District 5, 050663 Bucharest, Romania; violeta.rotarescu@fpse.unibuc.ro (V.Ș.R.); adrian.gorbanescu@unibuc.ro (A.G.)

**Keywords:** animal abuse, neglect, veterinary education, animal welfare, veterinary practice, veterinary communication

## Abstract

Animal abuse and neglect represent important challenges in companion-animal practice, with veterinarians playing a central role in their identification and management. This study investigated how Romanian companion-animal veterinarians recognise and manage suspected cases of abuse and neglect, and explored the relationships between professional training, familiarity with animal welfare legislation, and clinical practices. A cross-sectional survey was conducted using an original online questionnaire completed by 137 veterinarians. Descriptive statistics and inferential analyses were applied. Although 73% of respondents reported encountering suspected abuse cases, 93.4% said they had never received formal training in abuse identification, and 78.1% worked in practices without specific protocols for managing such cases. Reporting rates were low, with 71.5% of veterinarians never having submitted an official report. Neglect was identified as the most frequently encountered form of abuse. Significant associations were observed between familiarity with animal welfare legislation, level of training, and awareness of neglect, and veterinarians who perceived themselves as less prepared were more likely to express a need for additional education. These findings highlight critical gaps in veterinary training and institutional support, emphasising the need for structured educational programmes, lifelong learning and continuing professional development, standardised clinical procedures, and closer collaboration with authorities to improve animal welfare outcomes.

## Simple Summary

Veterinarians play a key role in identifying and reporting animal abuse; however, their effectiveness depends on training, legal awareness, and institutional support. This study assessed how Romanian companion-animal veterinarians recognise and manage suspected cases of abuse and neglect. An online questionnaire completed by 137 veterinarians revealed frequent exposure to suspected abuse cases but limited formal education and procedural support in managing them. Although topics related to animal abuse are addressed within veterinary forensic medicine curricula during university training, most respondents reported insufficient practical preparation for identifying, documenting, and reporting such cases in clinical practice. Most respondents reported lacking workplace protocols and specific training in abuse identification, and official reporting rates were low. Neglect was perceived as the most common form of cruelty. Statistical analyses showed significant associations between training, familiarity with legislation, and awareness of neglect. Veterinarians who felt less prepared expressed a greater need for additional education, while the presence of internal procedures was associated with greater transparency in clinical practice. These findings highlight the need for structured educational programmes and lifelong learning opportunities, alongside standardised protocols and stronger collaboration with authorities, to improve veterinarians’ ability to identify, document, and manage cases of animal abuse and neglect.

## 1. Introduction

The number of companion animals in Europe, particularly dogs and cats, has been steadily increasing, driving demand for improved medical care, the expansion of veterinary practices, and greater professional specialisation. A 2023 survey conducted by the European Commission reported that 84% of Europeans believe that farmed animals should benefit from stronger protection, whilst 74% stated that the welfare of companion animals also requires enhanced safeguarding [1]. In 2022, Europeans were estimated to own approximately 352 million pets, with 50% of households keeping at least one animal. Of these, 25% owned a dog and 27% a cat. In Romania alone, the pet population comprised 4.244 million dogs and 4.424 million cats [2].

In December 2023, the European Commission proposed the Regulation of the European Parliament and of the Council on the Welfare of Dogs and Cats and Their Traceability, which establishes minimum welfare requirements for these animals [3,4]. However, legislation alone—without parallel efforts in education and the promotion of responsible pet ownership—is unlikely to exert a significant and lasting impact on companion animal welfare across Europe [5]. Alongside education and the promotion of responsible ownership, effective enforcement mechanisms—including routine welfare inspections, auditing of compliance, and dedicated policing structures—are equally necessary to translate legislative provisions into tangible improvements in animal welfare [4,5].

There is a pressing need to move beyond the One Health framework towards the broader concept of One Welfare, which integrates animal husbandry conditions, workers’ welfare, and the environmental consequences of livestock farming. It also encompasses strategies to mitigate violence globally, highlights the critical connection between deficient animal and human welfare, and underscores the pivotal role that animal welfare plays in improving human well-being [6,7]. First articulated as a complement to One Health, One Welfare recognises that animal welfare, human well-being, and the environment are interdependent, and that improvements in one domain reinforce the others [6,7]. In companion-animal practice, this translates into concrete interconnections: the welfare of pets is shaped by the socio-economic circumstances and mental health of their owners [8], while cruelty towards animals frequently signals broader patterns of violence within households [9,10,11,12]. The framework therefore positions veterinarians not merely as providers of clinical care, but as sentinels at the interface between animal welfare and human welfare [7].

Awareness of the relationship between animal abuse or neglect and interpersonal violence is increasingly recognised and studied. To better understand this phenomenon and develop effective protocols, collaboration among diverse stakeholders is essential. These include veterinarians, physicians, animal welfare and forensic pathology specialists, animal protection services, police forces, local social services, legal professionals, and the academic community, to integrate and advance interdisciplinary knowledge [7,9,13].

In Romania, animal welfare is regulated by Law No. 205 of 26 May 2004, as revised and in force as of June 2024. The legislation stipulates that owners are responsible for their animals’ well-being and must ensure access to appropriate medical care. It also details the procedures that police officers must follow in cases of neglect or abuse [14]. There is no legal distinction between “intentional” and “non-intentional” acts; consequently, intent is considered irrelevant in the context of animal welfare. In the USA, several states require veterinarians to report suspected animal cruelty, recognising that such cases affect both animal welfare and public safety, because animal abuse is increasingly viewed as a potential indicator of interpersonal violence [15].

Battered pets, abused animals, or cases of non-accidental injury (NAI) have been the subject of scholarly investigation for several years. Veterinarians are often the first to identify signs of abuse. A 2001 study in the United Kingdom reported that NAI was a common finding in small animal practice, acknowledged by 91.3% of respondents, with 48.3% indicating that they had personally encountered such cases [16]. Similar findings were reported in Ireland, where 92.2% of survey participants recognised the occurrence of NAI and 44.3% reported encountering such cases in practice [17].

Recent research has adopted a broader perspective, incorporating sociodemographic factors, veterinarians’ professional experience, the prevalence of NAI, the number of abuse cases, and the veterinarian’s role in identifying and reporting such incidents [18]. The results are consistent globally. In the United States, 86% of veterinarians believed there to be a link between animal abuse and child abuse, whilst 77% perceived a link with spousal abuse. However, only 38.6% reported having received training to recognise animal abuse [19]. Another important aspect is that threats and abuse against animals are a predictor of domestic violence, both physical and sexual, as well as of other antisocial behaviours, including bullying and juvenile delinquency [10,20]. Victims of domestic violence reported that the companion animals with whom they have bonded tried to physically protect them from violent partners, but that the animals were also used as a form of control [11]. There is also a link between animal abuse and child abuse, as the former overlaps with other forms of abuse that children are exposed to [12].

In the specialised literature, the term animal abuse denotes maltreatment regardless of intent. The main categories identified include sexual, emotional, and physical abuse. Neglect, by contrast, is generally defined as the failure to provide basic welfare conditions or as abandonment [21,22]. Given the established connection between animal abuse and human violence, there is a strong need for enhanced education within the veterinary field [23]. Many veterinarians report lacking the resources to prevent abuse and consider themselves insufficiently trained to manage such cases and their legal implications [19]. At the same time, the field of veterinary forensics continues to evolve, offering an increasingly robust scientific foundation that informs best practices in evidence collection, forensic analysis, and record-keeping [24].

In the United States, practical resources, such as those provided by the American Veterinary Medical Association (AVMA), provide comprehensive support to practitioners. These materials offer conceptual clarification, guidance on clinical protocols and policies, tools for decision-making and risk assessment in suspected or confirmed cases of maltreatment, and references to the relevant legislation governing each stage [22].

Abuse against animals can be classified into several major categories such as active, passive (neglect), psychological, sexual and commercial exploitation [25]. In the present study, a distinction was drawn between neglect—understood as the failure to provide basic care, including food, water, grooming and veterinary attention—and inadequate welfare conditions, which refer to deficient housing and husbandry, such as inadequate shelter, permanent tethering on a short chain, or insufficient space and sanitation. Although both are passive forms of maltreatment and overlap conceptually, they were presented as separate categories in the questionnaire to mirror the wording of the Romanian legislation and the terminology used in everyday clinical practice [14].

Neglect cases are one of the most common forms of animal abuse encountered in veterinary practice. These include animals presenting a poor body condition, refusal of necessary medical treatment or grooming, refusal of euthanasia in cases where it is medically indicated, lack of concern for appropriate welfare conditions and cases involving animal hoarding, large ectoparasite burden, nails, hooves or horn overgrown, chronic pathologies unmanaged, dirty hair coat and dental disease [26,27,28,29].

The purpose of this study is to examine how Romanian veterinarians in companion animal practice address cases of animal abuse, including neglect. This analysis provides a basis for identifying educational needs, and gaps in procedural and institutional support that future intervention protocols and legislative refinements would need to address. Furthermore, it offers insights into how veterinarians engage with pet owners and identifies the most prevalent model of veterinarian–client communication—whether biomedical, bio–lifestyle–social, or consumerist [30,31,32]. Communication style and the transparency of clinical documentation were examined because they shape how welfare concerns are raised with owners, how neglect is documented in the medical record, and, ultimately, whether suspected abuse is escalated to the competent authorities. Collectively, these aspects have a profound impact on both animal and human welfare and may represent a critical step towards the One Welfare framework.

## 2. Materials and Methods

### 2.1. Study Design and Participants

This study employed a quantitative, cross-sectional design using a structured questionnaire administered to veterinarians legally authorised to practise in Romania. Participation was voluntary, and all respondents provided informed consent.

### 2.2. Questionnaire Development

An original questionnaire was designed using Google Forms (Supplementary Materials S1 and S2). The instrument consisted of twenty-five questions, structured into thematic sections. The first six items collected demographic information, including age, gender, location, type of practice, and years of professional experience. Sixteen questions explored issues related to animal abuse and neglect, such as familiarity with relevant legislation, professional training, internal procedures, and reporting practices. Two additional questions examined the veterinarian–client relationship model (biomedical, bio–lifestyle–social, or consumerist), and the final question was open-ended, allowing respondents to share comments or reflections on the topic. The questionnaire was distributed online via professional Facebook groups and veterinary community platforms between 2 and 19 December 2024.

### 2.3. Data Collection

Responses were collected electronically and automatically stored via Google Forms. No personal identifiers were collected to ensure respondent confidentiality.

### 2.4. Data Analysis

Data were exported to IBM SPSS Statistics, Version 30 (IBM Corp., Armonk, NY, USA), for both descriptive and inferential analyses. Descriptive statistics were used to summarise demographic and categorical variables. Inferential analyses were conducted to examine associations between variables: Chi-square (χ^2^) tests were applied to assess relationships between categorical variables, while correlation analyses (Pearson’s *r* and Spearman’s ρ) were used to evaluate relationships between ordinal and continuous variables. Because several items used five-point Likert-type scales and produced sparse contingency tables, response categories were collapsed for the inferential tests into three levels (1–2, 3, and 4–5), and age into three groups (24–30, 31–40, and over 40 years), so that the expected cell-count requirements of the χ^2^ test were met; full five-level distributions are reported descriptively in the tables. Effect sizes are reported as Cramér’s V. A sensitivity analysis indicated that the sample (*N* = 137) allowed the detection of medium-sized effects (w ≥ 0.27 for df = 2; w ≥ 0.30 for df = 4) with 80% statistical power at α = 0.05. The level of statistical significance was set at *p* < 0.05.

### 2.5. Ethical Considerations

Participation was entirely voluntary and anonymous. The study adhered to the ethical principles of confidentiality, data protection, and informed consent, in accordance with the standards for research involving human participants as defined by the European Code of Conduct for Research Integrity.

### 2.6. AI-Assisted Tools

Generative AI tools (ChatGPT, OpenAI GPT-4.5; Claude Opus 4.8, Anthropic) were used during the preparation of this manuscript. Their use included language and readability editing; assistance with drafting and reorganising portions of the text; the generation of tables; and cross-checking of statistical calculations and support for interpreting the results. Generative AI was not used for the study design, data collection, or the generation of original research data. All AI-generated suggestions were critically reviewed and revised by the authors, who take full responsibility for the accuracy, validity, and integrity of the content.

## 3. Results

### 3.1. Demographic Characteristics of Respondents

A total of 137 veterinarians voluntarily completed the questionnaire. Of these, 62% (*N* = 85) were employed in clinics or companion animal practices, 17.5% (*N* = 24) in veterinary hospitals, and 16.8% (*N* = 23) in practices treating both companion and farm animals. The remaining five respondents (one each) worked in the Food Safety and Veterinary Department, academia (pathological anatomy), veterinary product distribution, a rural practice, and an Animal Protection Bureau.

As shown in Table 1, nearly half of the respondents were aged 24–30 years, followed by 37.2% aged 31–40 years, indicating that most participants were early-career veterinarians. In terms of gender, 72.3% (*N* = 99) identified as female and 27.7% (*N* = 38) as male. This distribution is consistent with the ongoing feminisation of the veterinary profession in Europe, which is most pronounced among younger age cohorts [33,34], and should be interpreted in light of the young age profile of the present sample, as official statistics on the gender structure of the veterinary profession in Romania are not publicly available.

Most respondents (78.1%, *N* = 107) worked in urban areas, 12.4% (*N* = 17) in rural areas, and 9.5% (*N* = 13) in both urban and rural areas. In total, the participants represented 26 of Romania’s 42 counties. Over half of the respondents were based in Bucharest, while the remainder were distributed across 25 other counties, reflecting a reasonably broad geographic spread (Table 2).

Nearly half of the participants (46.7%) had less than five years of experience, confirming that the sample was dominated by young professionals (Table 3).

### 3.2. Familiarity with Legislation and Training

In Romania, as in other European countries, each professional field is regulated by legislation. Self-assessed familiarity with the legislation on animal abuse was measured on a five-point Likert-type scale ranging from 1 (no knowledge) to 5 (very familiar). Responses spanned the full scale: 38.7% (*N* = 53) reported moderate knowledge, 17.5% (*N* = 24) a good understanding, and 18.2% (*N* = 25) described themselves as very familiar, whereas 20.4% (*N* = 28) were only somewhat familiar and 5.1% (*N* = 7) reported no knowledge (Table 4).

Most veterinarians (93.4%, *N* = 128) had never received training in identifying or managing cases of abuse, while only 6.6% (*N* = 9) had participated in such training. A Chi-square test of association revealed a significant relationship between training level and familiarity with legislation, χ^2^(2, *N* = 137) = 7.89, *p* = 0.019, Cramér’s V = 0.24. This result indicates that participation in training is positively associated with greater familiarity with the legislation regulating abuse. Nevertheless, because only nine respondents had received such training, this association should be interpreted with caution.

### 3.3. Procedures, Reporting, and Suspected Cases

**Figure 1:**
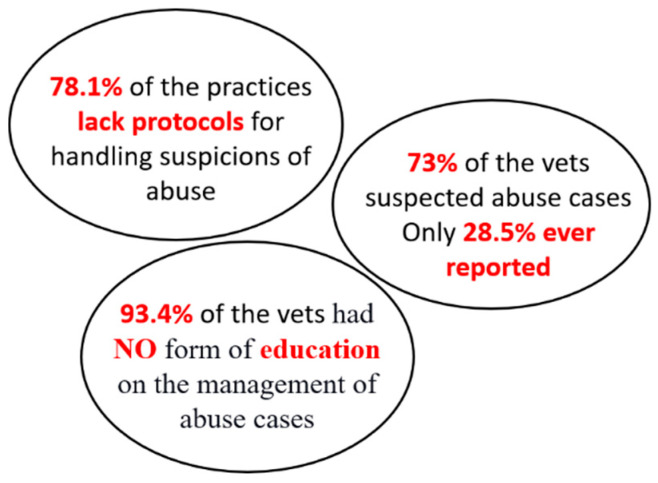
Realities of Romanian veterinary practice regarding suspicion of abuse and reporting.

Most practices (78.1%, *N* = 107) lacked formal procedures for handling suspected abuse, while 21.9% (*N* = 30) had protocols in place (Figure 1).

A total of 73% (*N* = 100) of veterinarians reported encountering cases in which abuse was suspected at some point in their careers, whereas 27% (*N* = 37) had never suspected such a case. Referring specifically to the previous 12 months, 47.4% (*N* = 65) had suspected between one and five cases, 11.7% (*N* = 16) five to ten, 4.4% (*N* = 6) ten to twenty, and 1.5% (*N* = 2) more than twenty cases, while 35% (*N* = 48) had suspected none during this period. Although most respondents had thus encountered suspected abuse at some point in their career, only 28.5% (*N* = 39) had ever submitted a formal report to the relevant authorities, while 71.5% (*N* = 98) had never done so. Notably, this low reporting rate cannot be explained by respondents not knowing whom to contact, as 83.9% (*N* = 115) stated that they knew to which institution suspected cases should be reported. Taken together, the data in Figure 1 outline a consistent pattern: suspicion of abuse is widespread and the reporting pathway is known, yet formal reporting remains the exception, in a context in which almost four out of five practices lack internal procedures for such cases.

### 3.4. Types of Abuse and Familiarity with Neglect

Neglect was identified as the most common form of abuse (Table 5), reported by 36.5% (*N* = 50) of respondents. This was followed by inadequate welfare conditions (23.4%, *N* = 32), physical abuse (21.9%, *N* = 30), abandonment (12.4%, *N* = 17), and premature separation of the mother from her offspring before eight weeks of age (3.6%, *N* = 5). The remaining three respondents (2.2%) indicated other forms, such as organised animal fighting or combinations of the listed categories.

Regarding official reports, 81.8% (*N* = 112) of veterinarians had never completed any official documentation (e.g., consultation records or written reports) formally describing a suspected case of abuse, whereas only 18.2% (*N* = 25) reported having done so.

When asked to rate their level of training in identifying abuse and completing the associated documentation, most veterinarians reported limited proficiency: 36.5% (*N* = 50) indicated no training, 18.2% (*N* = 25) minimal training, 27.7% (*N* = 38) moderate training, 11.7% (*N* = 16) sufficient training, and 5.8% (*N* = 8) extensive training.

A chi-square test of association revealed a statistically significant relationship between familiarity with animal abuse legislation and the level of training in identifying and documenting suspected cases, χ^2^(4, *N* = 137) = 27.76, *p* < 0.001. The strength of the association, indicated by Cramér’s V = 0.32, suggests a moderate positive relationship: veterinarians with greater legislative awareness also reported higher levels of training in identifying and documenting abuse.

The primary reason cited by most veterinarians for reporting abuse to the authorities was the protection of the animal, reported by 89.1% (*N* = 122). Ethical considerations ranked second (38%, *N* = 52), followed by adherence to the professional code of conduct (37.2%, *N* = 51). Legal implications were mentioned by 18.2% (*N* = 25), while 8.8% (*N* = 12) reported the intention to safeguard household members. A small minority (2.2%, *N* = 3) stated that they would report abuse solely because workplace policies required it.

In cases of suspected abuse, 58.4% (*N* = 80) of veterinarians indicated they would inform the owner of their intention to report, whereas 39.4% (*N* = 54) said they would not. Three respondents adopted alternative approaches: one would inform the owner only if the owner was unaware of the abuse, one stated that the decision would depend on the specific details of each case, and one was unsure how they would proceed.

Regarding abuse through negligence, most veterinarians reported at least some familiarity with the topic: 41.6% (*N* = 57) described themselves as moderately familiar, 18.2% (*N* = 25) as having a good understanding, and 19% (*N* = 26) as very familiar. Only 5.8% (*N* = 8) reported having no knowledge of the subject, while 15.3% (*N* = 21) reported poor familiarity (Table 6).

A chi-square test of association revealed a statistically significant relationship between respondents’ familiarity with legislation regulating animal abuse and their familiarity with the concept of neglect as a form of abuse, χ^2^(4, *N* = 137) = 46.77, *p* < 0.001 (Table 7). The strength of the association, measured by Cramér’s V = 0.41, indicates a moderate to strong relationship. Correlation analyses further supported these findings, with Pearson’s r = 0.46 (*p* < 0.001) and Spearman’s ρ = 0.47 (*p* < 0.001), confirming a positive association between the two variables.

These results suggest that veterinarians who are more familiar with legislation regulating animal abuse also tend to demonstrate a greater awareness and understanding of neglect as a form of abuse. This finding highlights the interdependence between legal knowledge and professional competence: a solid understanding of the legal framework appears to enhance recognition of neglect, while increased awareness of neglect may, in turn, foster greater engagement with the relevant legislation.

Within the category of neglect, respondents identified several specific manifestations. The most frequently reported issue was untreated acute or painful medical conditions, accounting for 38.7% (*N* = 53) of cases. This was followed by untreated chronic illnesses at 19.7% (*N* = 27) and emaciation at 19% (*N* = 26). Less frequently mentioned were cases involving animal hoarding (5.8%, *N* = 8) and obesity (3.6%, *N* = 5). The remaining eighteen respondents (13.1%) indicated combinations of the listed issues, fifteen of whom stated that all of them should be considered relevant examples of neglect.

### 3.5. Education and Training Needs

Both formal and informal education are essential for practitioners and pet owners alike. In this study, 85.4% (*N* = 117) of veterinarians reported having encountered situations in which they educated pet owners about issues classified as neglect, such as obesity, malnutrition, or untreated chronic medical conditions. A small proportion (5.1%, *N* = 7) reported never having faced such situations, while 9.5% (*N* = 13) indicated uncertainty. The vast majority of respondents (82.4%, *N* = 112 of valid responses; one respondent did not answer this item) expressed a need for additional training to improve their ability to identify and manage cases of abuse.

A chi-square test of association revealed a statistically significant relationship between veterinarians’ self-assessed level of training in identifying and documenting abuse cases and their perceived need for additional training, χ^2^(2, *N* = 136) = 13.14, *p* = 0.001, Cramér’s V = 0.31 (Table 7). Those who rated their training level as lower were more likely to express a desire for further education. In contrast, respondents with higher self-assessed competence were less likely to indicate such a need.

### 3.6. Veterinarian–Client Communication Patterns

The analysis of communication styles revealed that most veterinarians preferred a collaborative approach. A substantial majority, 83.9% (*N* = 115), reported viewing pet owners as partners with whom they work collaboratively. This approach aligns with the bio–lifestyle–social communication pattern. In contrast, 11.7% (*N* = 16) of respondents described a more authoritarian style, characterised by a stronger adherence to their own professional opinion and less consideration of client preferences. Only 4.4% (*N* = 6) reported a consumerist approach, in which the client’s expectations and choices primarily guide clinical decisions.

At the end of an appointment, 60.6% (*N* = 83) of practices reported providing pet owners with a copy of the medical record, either in physical or electronic form. By comparison, 35.8% (*N* = 49) indicated that no copy was provided, while 3.6% (*N* = 5) were uncertain whether such a procedure existed in their workplace.

A chi-square test of association identified a statistically significant relationship between the veterinarian’s age and the likelihood that the owner would receive a copy of the consultation record, χ^2^(4, *N* = 137) = 19.72, *p* < 0.001, Cramér’s V = 0.27 (Table 7), indicating a moderate association. Veterinarians aged 24–30 years reported providing a copy more frequently than expected, while those aged 40 years and above reported not providing a copy more often than expected. No substantial deviations were observed for the “Not sure” response category. Additionally, a statistically significant association was found between the practice environment and whether the owner receives a copy of the consultation record, χ^2^(4, *N* = 137) = 11.14, *p* = 0.025, Cramér’s V = 0.20, indicating a small-to-moderate association. Veterinarians practising in urban environments reported providing a copy more frequently than expected, whereas those in rural environments reported not providing a copy more often than expected. For veterinarians working in both environments, responses were more evenly distributed, with a predominance of “Yes” responses.

A significant association exists between the presence of workplace procedures regarding abuse and whether the owner receives a copy of the consultation record, χ^2^(2, *N* = 137) = 6.32, *p* = 0.042, Cramér’s V = 0.21 (Table 7). This finding suggests that the presence of internal procedures influences whether owners receive a copy of the medical record, although the strength of the association was weak. All statistically significant associations identified in this study, together with their effect sizes, are summarised in Table 7.

## 4. Discussion

The results of this study highlight several important aspects of Romanian veterinarians’ engagement with animal welfare, particularly in the recognition, reporting, and management of cases of abuse and neglect. Despite Romania’s alignment with European animal welfare legislation, the findings reveal notable gaps in both training and procedural implementation across veterinary practices.

A notable first observation is the low level of formal training in identifying and managing abuse cases. Topics related to physical and sexual abuse have been included in forensic pathology curricula for approximately two decades [35]; nevertheless, most respondents (93.4%) reported never having participated in any dedicated training in this field. At the same time, 85.4% of respondents reported educating pet owners about issues commonly classified as neglect. This finding suggests that although veterinarians possess knowledge of animal welfare and neglect, they may not always recognise these competencies within the broader framework for identifying and managing animal abuse. This apparent discrepancy may reflect not only the limited availability of specialised postgraduate training, but also a lack of awareness regarding the relevance of abuse-related content acquired during undergraduate education. Consequently, knowledge acquired during veterinary training may not consistently translate into the practical skills required to recognise, document, and report cases of abuse. Similar situations were reported in Brazil and Colombia, where vets described insufficient training in veterinary forensics and animal welfare, and only a small minority reported suspected NAI to the authorities [36]. Australian vets have also identified the need for additional education and professional support to effectively recognise and respond to suspected or confirmed cases of animal abuse [37]. Collectively, these findings emphasise the importance of strengthening both continuing professional development and awareness of existing competencies in order to improve veterinarians’ preparedness to manage abuse cases effectively.

Participation in educational activities enhances awareness of legal obligations and responsibilities so that veterinarians who have attended training are also more familiar with the legal framework (*p* < 0.05). Also, cruelty training and a relationship with law enforcement directly influence the number of suspected cases identified and reported [38].

Veterinarians who are more knowledgeable about legislation regulating animal abuse also tend to report a higher level of training and competencies in recognising and documenting abuse (*p* < 0.001). This association highlights the interdependence between legal awareness and professional practice. Familiarity with the legislative framework appears to enhance veterinarians’ ability to identify cases of animal cruelty, interpret them correctly, and follow appropriate procedural steps. Conversely, practical training in recognising signs of abuse may encourage greater interest in understanding the relevant laws, creating a reciprocal learning effect.

At the same time, the majority of veterinary practices (78.1%) lack procedures for managing suspected abuse cases. Even though 73% of respondents reported having encountered situations of suspected abuse, only 28.5% had submitted an official report. This mismatch between suspicion and reporting highlights the uncertainty surrounding professional responsibilities and the absence of clear procedural guidance. While the establishment of the Animal Police in Romania in 2020 represented an important legislative advancement, this study’s findings suggest that collaboration between veterinarians and law enforcement authorities remains limited in everyday practice. Such attitudes may be influenced by limited exposure to reporting practices during both undergraduate education and continuing professional development and lifelong learning programmes. Or it might be similar to the situation in South Korea, where vets believe that the current legal and police systems cannot ensure security and welfare, following the reports [39]. The lack of well-defined procedures discourages reporting, increases professional hesitation, and reduces the effectiveness of the existing legal framework. Meanwhile, suspected cases are more likely to be reported by those who indicated an existing workplace policy in that direction [38]. Veterinarians may feel more comfortable managing welfare concerns through client education than through formal involvement of the authorities. Also, well-structured lifelong learning programmes that ensure a quality educational background may contribute not only to closing this competency gap, but also to promoting business enhancement within the veterinary sector, by equipping practitioners with the confidence and procedural knowledge needed to integrate good professional practice into their daily activity [40].

The gap between the 73% of respondents who had suspected abuse and the 28.5% who had ever reported it warrants closer interpretation, particularly as the low reporting rate cannot be attributed to a lack of knowledge of the reporting pathway: 83.9% of respondents stated that they knew which institution to contact. Several overlapping barriers are likely to operate. First, in the absence of internal protocols—lacking in almost four out of five practices—the decision to report rests entirely on the individual clinician; consistent with this, suspected cases have been shown to be more likely to be reported where a workplace policy for handling them exists [38]. Second, the diagnostic and evidentiary threshold may deter formal reporting: most respondents had received no training in identifying or documenting abuse, and a lack of knowledge, resources and experience has elsewhere been identified as a barrier to reporting [18], a difficulty compounded in Romania by a legal framework that does not distinguish intentional from non-intentional harm [14]. Third, there are relational and economic disincentives: reporting a client may rupture the veterinarian–client relationship and lead to the loss of clients, with a potential impact on practice income, and veterinarians may be uncertain whether a report would be handled effectively [18,41]. It is also plausible that some clinicians judge they can do more for the animal by educating and supporting the owner than by initiating a report of uncertain benefit [18,38]. Finally, confidence in the enforcement system matters: although an Animal Police unit was established in Romania in 2020, its collaboration with veterinary practitioners appears limited in everyday practice, echoing the South Korean experience, where veterinarians doubted that the legal and police systems could ensure animal welfare and security following a report [39]. Where practitioners anticipate that a report will generate professional risk without securing protection for the animal, reporting is likely to remain the exception rather than the norm.

These findings also invite reflection on a question this study, like much of the literature, has largely taken for granted: what role veterinarians should play in reporting abuse and neglect. The assumption that reporting is always the appropriate response is not self-evident. Veterinarians occupy a dual position as advocates for animal welfare and as professionals bound by a relationship of trust with their clients—a tension reflected in the concerns about client confidentiality that veterinarians report when deciding whether to act [18]. Whereas some jurisdictions place veterinarians under a mandatory duty to report suspected cruelty [15], others do not; in Romania, veterinarians do not appear to be subject to such a statutory obligation, so the decision to report is discretionary rather than obligatory. A defensible position is that the veterinarian’s primary obligation is to the welfare of the animal, which in some cases is best served by reporting to the authorities, but in others by client education, closer follow-up, or facilitating access to care, reserving formal reporting for situations involving deliberate cruelty, recurrence, or a risk to human members of the household, given the recognised link between animal and interpersonal violence [9]. Making this reasoning explicit—rather than treating reporting as an unexamined default—would help the profession develop proportionate, welfare-centred guidance and would clarify the ethical and legal expectations placed on practitioners.

Neglect was identified as the most common form of cruelty (36.5%), followed by inadequate welfare conditions, physical abuse, and abandonment. Within the broader category of neglect, veterinarians most frequently observed acute, untreated medical problems (38.7%), untreated chronic diseases (19.7%), and emaciation (19%). These data show that neglect is not always linked to deliberate acts but often results from a lack of education and awareness among owners, and, in some cases, from limited resources rather than ill intent. Financial hardship, the absence of affordable or accessible veterinary services, and the lack of means of transport to a practice may all prevent owners from meeting their animals’ basic needs, so that what presents clinically as neglect may reflect socio-economic constraints as much as indifference [42]. Distinguishing these situations is important, because they call for different responses—support, referral, and education rather than sanction. Insufficient basic maintenance and care of companion animals was also the primary form of non-compliance with the welfare legislation in Finland [4]. Preventive action should focus on education; owners should be educated about the basic needs of their companions [25].

Veterinarians who better understand the legal framework are also more familiar with neglect as a form of abuse and aware of its indicators (*p* < 0.001). These correlations emphasise that once a learning pattern is established, it strengthens knowledge on multiple levels—both legal and practical—creating a positive educational loop between theory and applied recognition. Midwestern University College of Veterinary Medicine developed the standardised client scenario “Grizabella’s Final Fight” to help students gain confidence in discussing animal cruelty while also using Calgary–Cambridge communication skills to manage and de-escalate conflict during such discussions. The model is intended to be adaptable by other veterinary schools, allowing students to practise cruelty-reporting conversations within the framework of their own state-specific legislation and professional ethics [43]. Beyond isolated scenarios of this kind, the challenge is how to engage both students and practising veterinarians with the topic in a sustained way. Abuse recognition has historically received little curricular time [44]. Rather than treating it as a niche forensic subject, curricula could embed it within ethics teaching that makes explicit the veterinarian’s role at the animal–human welfare interface, combining case-based discussion, simulated client encounters such as those trialled in veterinary education [43] and clear guidance on legal duties and referral pathways. For qualified veterinarians, short continuing-education modules, workplace protocols, and accessible reference materials—analogous to the AVMA resources available in the United States [22]—would help translate awareness into confident practice, consistent with evidence that training and workplace policies are associated with greater recognition and reporting [38]. Framing this as a core professional and ethical responsibility, rather than an optional interest, may be the most effective way to increase both recognition and appropriate reporting.

The relationship between self-assessed training level and the perceived need for additional education was also analysed. Veterinarians who considered their training insufficient were more likely to request further instruction (*p* < 0.05), whereas those with higher self-assessed competence reported a lower need for additional training. This correlation reflects realistic self-evaluation and highlights the necessity of continuous professional education. Targeted training programmes could therefore increase competence, standardise evaluation and reporting methods, and reduce uncertainty in practical situations. This highlights the role of self-assessment as a useful indicator for identifying areas where lifelong education and continuing professional development are most needed within the veterinary community. This interpretation should nevertheless be tempered by a well-recognised limitation of self-report: self-assessed knowledge does not always correspond to actual competence. Across the health professions, confidence and measured ability are often only weakly related, and individuals with the largest knowledge gaps tend to be the least able to recognise them [45,46]. Self-reported familiarity with legislation may therefore index confidence as much as demonstrated legal knowledge. Consequently, the associations observed here between perceived legal familiarity, self-rated training, and awareness of neglect should be interpreted with caution, and objective assessments of legal and forensic competence would be a valuable complement to self-report in future studies.

A significant number of veterinarians (82.4%) expressed the need for additional training to improve their ability to identify and report abuse. This widespread demand supports the idea that professional development in this area remains underrepresented in continuing education. Similarly, the relationship between legal knowledge, awareness of neglect, and perceived competence suggests that education must integrate both theoretical and practical components to have measurable effects.

Communication with clients also plays an essential role in identifying neglect and ensuring compliance with welfare standards [30,32]. The results show that most veterinarians (83.9%) perceive their relationship with pet owners as collaborative, reflecting a bio–lifestyle–social communication pattern, and that only 35.8% of clinics did not provide copies of medical records. These findings represent an improvement over a previous study, in which 57.3% (*N* = 98) of pet owners reported not receiving a copy of their animal’s medical file [47]. Emotional intelligence plays an important role in companion animal medicine, as most pet owners consider their pets family members; therefore, the emotional content of conversations and decisions about their pets’ health care has increased [48]. Also, educated pet owners can be more careful and avoid neglectful behaviours [28].

This approach supports mutual understanding and shared responsibility for animal welfare. However, a smaller proportion of veterinarians (11.7%) described an authoritarian approach, and 4.4% described a consumerist model in which the owner’s opinion prevails. These findings indicate diversity in professional interaction styles, which may influence how abuse and neglect are discussed, recognised, and addressed. This link is directly relevant to the central questions of the present study. A collaborative, relationship-centred style—already associated with greater client engagement and adherence to veterinary recommendations [49]—is likely to make it easier for veterinarians to raise sensitive welfare concerns, to educate owners about neglect, and, where necessary, to broach the possibility of reporting, whereas a purely authoritarian or consumerist dynamic may discourage such conversations. Communication style is therefore not a peripheral issue but part of the practical toolkit through which abuse and neglect are recognised and managed.

The data also revealed differences related to the transparency of clinical documentation. At the end of consultations, 60.6% of practices provided clients with a copy of the medical record, while 35.8% did not. Providing clear and accessible medical documentation contributes to owner compliance and trust [49,50].

The significant association between age and the provision of a copy of the medical record suggests meaningful generational differences in documentation practices (*p* < *0*.05). Younger veterinarians (24–30 years) were considerably more likely to provide owners with a copy of the consultation record, which may reflect recent curricular changes, increased exposure to digital documentation systems, and a stronger emphasis on transparency and client-centred communication during their training. In contrast, veterinarians aged 40 years and above were more likely to report not offering such documentation or being uncertain whether this practice was implemented in their clinical environment. These results indicate that documentation practices vary across age groups and reinforce the need for standardised protocols to enhance consistency, transparency, and continuity of care in veterinary settings.

Access to resources and workplace infrastructure may influence documentation practices, as suggested by the association between practice environment and the provision of a copy of the medical record. Veterinarians working in urban clinics were considerably more likely to provide owners with a copy of the consultation record (*p* < *0*.05). This pattern may reflect greater digitalisation, stronger administrative support, and the routine use of electronic medical records in urban practices. In contrast, veterinarians working in rural settings were more likely to report that they did not offer such documentation, possibly due to limited equipment, reliance on handwritten notes, reduced staffing, or differing client expectations. Those practising in both environments displayed an intermediate pattern, suggesting that mixed exposure may encourage more standardised approaches. Overall, these findings highlight how structural and environmental factors can influence transparency and communication practices in veterinary care, reinforcing the need for uniform protocols across practice settings, similar to findings in Thailand, Bulgaria and Turkey [51,52].

### Limitations

Several limitations apply. The sample was small (*N* = 137) and self-selected through an online convenience survey, over-representing early-career veterinarians and those based in Bucharest; the findings are therefore not representative of the profession nationally, and the reported proportions should be read as indicative rather than as population estimates. All data were self-reported and thus subject to social-desirability and recall bias, and self-assessed familiarity with legislation may reflect confidence rather than demonstrated knowledge; the questionnaire was also original and not formally validated. The cross-sectional design allows associations to be described but not causal inference. Finally, Likert categories were collapsed to meet the assumptions of the χ^2^ test, some associations rested on small subgroups (notably the nine respondents with formal training), and multiple tests were run without correction for multiplicity. Despite these limitations, the study adds to the limited evidence on how Romanian companion-animal veterinarians manage suspected abuse and neglect and provides a basis for larger, longitudinal research.

## 5. Conclusions

Overall, the findings of this study indicate that Romanian veterinarians recognise the occurrence of abuse and neglect, but face substantial systemic barriers in addressing these cases effectively. The absence of formalised procedures in most practices, limited access to specialised training, and insufficient collaboration between veterinary services and relevant authorities diminish the effectiveness of existing animal welfare mechanisms.

Significant associations were observed between training, familiarity with legislation, and recognition of neglect, indicating that legal knowledge and professional competence develop in parallel.

These findings underscore the importance of strengthening veterinary curricula and lifelong learning to incorporate current advances in veterinary forensics and animal welfare science, which is essential for improving both the identification and the management of abuse cases. Establishing standardised protocols and strengthening cooperation with relevant authorities would enhance the detection, documentation, and reporting of abuse.

For veterinary faculties, this implies moving abuse and neglect from a brief forensic-pathology topic to a theme integrated across the curriculum: teaching the signs of non-accidental injury and neglect alongside their clinical differentials, the relevant legislation (Law No. 205/2004) and reporting procedures, and the basics of forensic documentation, ideally through case-based and simulated client encounters such as the “Grizabella’s Final Fight” scenario. Embedding these within veterinary ethics teaching, and extending them to practising clinicians through short continuing-education modules and workplace protocols, would help close the gap between university knowledge and confident action in practice.

Enhancing communication with pet owners and ensuring transparent documentation would further strengthen trust and compliance, while supporting veterinarians in fulfilling their ethical and legal obligations. Such measures would not only advance animal welfare in Romania but also align veterinary practice with a broader approach that recognises the interdependence of animal well-being, human welfare, and social responsibility.

## Figures and Tables

**Table 1:** Age distribution of respondents.

Age Group	Respondents (*N*)	Percentage (%)
24–30 years	64	46.7
31–40 years	51	37.2
41–50 years	15	10.9
51–60 years	5	3.6
Over 60 years	2	1.5

**Table 2:** Geographic distribution of respondents by county.

Respondents (*N*)	Counties	Percentage (%)
70	Bucharest	51.1
8	Iaşi	5.8
7	Ilfov	5.1
6	Argeş	4.4
5	Vâlcea	3.6
4	Constanţa	2.9
3	Braşov, Neamţ, Suceava, Teleorman, Timiş	11.0
2	Bacău, Buzău, Dâmboviţa, Galaţi, Giurgiu, Prahova, Vrancea	10.5
1	Alba, Dolj, Gorj, Maramureş, Mehedinţi, Satu Mare, Sibiu, Tulcea	5.6

**Table 3:** Professional experience of respondents.

Years of Experience	Respondents (*N*)	Percentage (%)
1–5 years	64	46.7
6–10 years	36	26.3
11–20 years	24	17.5
21–30 years	8	5.8
Over 30 years	5	3.6

**Table 4:** Familiarity with legislation regulating animal abuse and neglect.

Familiarity Level	Respondents (*N*)	Percentage (%)
No knowledge	7	5.1
Somewhat familiar	28	20.4
Moderate knowledge	53	38.7
Good understanding	24	17.5
Very familiar	25	18.2

**Table 5:** Veterinarians’ opinions on the most common forms of abuse.

Form of Abuse	Respondents (*N*)	Percentage (%)
Neglect	50	36.5
Inadequate welfare conditions	32	23.4
Physical abuse	30	21.9
Abandonment	17	12.4
Premature separation of offspring	5	3.6
Other forms	3	2.2

**Table 6:** Veterinarians’ self-reported familiarity with neglect as a form of abuse.

Familiarity Level	Respondents (*N*)	Percentage (%)
No knowledge	8	5.8
Poor familiarity	21	15.3
Moderate knowledge	57	41.6
Good understanding	25	18.2
Very familiar	26	19

**Table 7:** Summary of statistically significant associations and their effect sizes.

Association	χ^2^ (df)	*p*	Effect Size/Correlation
Training received × familiarity with legislation	7.89 (2)	0.019	V = 0.24
Familiarity with legislation × level of training	27.76 (4)	<0.001	V = 0.32
Familiarity with legislation × familiarity with neglect	46.77 (4)	<0.001	V = 0.41; r = 0.46; ρ = 0.47
Level of training × perceived need for training	13.14 (2)	0.001	V = 0.31
Age × provision of a copy of the record	19.72 (4)	<0.001	V = 0.27
Practice environment × provision of a copy of the record	11.14 (4)	0.025	V = 0.20
Workplace procedures × provision of a copy of the record	6.32 (2)	0.042	V = 0.21

All χ^2^ tests used response categories collapsed as described in Section 2.4 (*N* = 137, except the training-need association where *N* = 136). V = Cramér’s V; r = Pearson’s correlation; ρ = Spearman’s correlation.

## Data Availability

The original contributions presented in this study are included in the article/Supplementary Material. Further inquiries can be directed to the corresponding author(s).

## Supplementary Materials

The following supporting information can be downloaded at https://www.mdpi.com/article/10.3390/vetsci13070696/s1. Supplementary Material S1, Original Romanian Version of the Questionnaire Used in the Study. Supplementary Material S2, English Translation of the Questionnaire Used in the Study. Supplementary Material S3, Ethics Committee Approval. Supplementary Material S4, Raw database.
