# Supplementary material for: Animal Abuse and Neglect in Companion-Animal Practice: The Role of Training, Legislation, and Veterinarian–Client Relationships in Romania
Source: Vet Sci. 2026 Jul 17;13(7):696. doi: 10.3390/vetsci13070696 (PMC13418691; doi:10.3390/vetsci13070696)
Supplement: Supplementary file 1 [file vetsci-13-00696-s001.zip › Supplementary Material S1 Original Romanian Version of the Questionnaire Used in the Study.pdf]

Mod de previzualizare

Publicat

Copiați linkul respondentului

Acest formular nu acceptă răspunsuri.

Gestionează setările de publicare

Chestionar adresat medicilor veterinari - Analiză a gestionării cazurilor de abuz împotriva animalelor de companie cu proprietar.

Chestionarul este adresat medicilor veterinari cu drept de liberă practică pe teritoriul României. Are ca scop colectarea de date necesare unui studiu care analizează modalitatea de gestionare a cazurilor de abuz, inclusiv a abuzului prin neglijență, împotriva animalelor de companie cu proprietar.

Vă rugăm să aveți în vedere experiențe proprii, pe care le-ați întâlnit în practică.
Nu colectăm și nu utilizăm date cu carcater personal.

Datele oferite vor fi stocate și analizate pe platforma Google Drive, care a întreprins acțiunile necesare pentru a se alinia cerințelor Regulamentului (UE) 2016/679 privind protecţia persoanelor fizice în ceea ce privește prelucrarea datelor cu caracter personal și privind libera circulaţie a acestor date și de abrogare a Directivei 95/46/CE (Regulamentul general privind protecţia datelor).
Echipa de cercetare este mixtă profesor coordonator, medic veterinar și psiholog, doctorand al Facutății de Medicină Veterinară, USAMV București. Datele obținute vor fi publicate sub forma unei lucrări de doctorat.

* Indică o întrebare obligatorie

Mod de previzualizare

Publicat

Copiați linkul respondentului

Acest formular nu acceptă răspunsuri.

Gestionează setările de publicare

- Cabinet/clinică veterinară pentru animale de companie și animale de rentă
- Spital veterinar dedicat animalelor de companie
- Altele:

*

Vârstă:

- 24-30 ani
- 30-40 ani
- 40-50 ani
- 50-60 ani
- Peste 60 de ani

Profesați în mediu: *

- Urban
- Rural
- Ambele

În ce județ vă desfășurați activitatea? *

Alege

Mod de previzualizare

Publicat

Copiați linkul respondentului

Acest formular nu acceptă răspunsuri.

Gestionează setările de publicare

- 1-5 ani
- 6-10 ani
- 11-20 ani
- 21- 30 ani
- Peste 30 de ani

Vă identificați drept: *

- Femeie
- Bărbat
- Non-binar
- Altele

Cât de familiarizat sunteți cu legislația care reglementează abuzul împotriva animalelor? *

	1	2	3	4	5	
Nu cunosc deloc acest subiect	○	○	○	○	○	Foarte familiarizat

Mod de previzualizare

Publicat

Copiați linkul respondentului

Acest formular nu acceptă răspunsuri.

Gestionează setările de publicare

Da

Nu

Aveți (la locul de muncă) proceduri care să fie aplicate în cazurile de suspiciune de abuz? *

Da

Nu

Știți cărei înstituții trebuie să raportați suspiciunile de cazuri de abuz? *

Da

Nu

V-ați întâlnit cu cazuri în care să suspicionați abuz împotriva animalelor? *

Da

Nu

Mod de previzualizare

Publicat

Copiați linkul respondentului

Acest formular nu acceptă răspunsuri.

Gestionează setările de publicare

- Niciun caz
- Între 1-5 cazuri
- 5-10 cazuri
- 10-20 cazuri
- Peste 20 cazuri

Care credeți că este cea mai frecventă formă de abuz împotriva animalelor? *

- Abuz fizic (rezultat în urma violenței fizice, a loviturilor, utilizării de arme albe, spânzurării/ ștrangulării, arsuri, intoxicații, etc.)
- Abuz sexual
- Abuz prin neglijență
- Abuz prin organizarea de lupte între animale
- Abandonarea și sau alungarea animalelor
- Despărțirea mamelor de pui înainte de împlinirea vârstei de 8 săptămâni
- Neasigurarea condițiilor de bunăstare (adăpost neadecvat, lanț scurt (mai mic de 2 metri), padoc de mici dimensiuni, harnă și apă necorespunzătoare, etc.)
- Altele:

Mod de previzualizare

Publicat

Copiați linkul respondentului

Acest formular nu acceptă răspunsuri.

Gestionează setările de publicare

Da

Nu

Ați completat vreodată un raport/ fișă de consultație/ document oficial privind o situație de abuz, unde să descrieți clar abuzul suspicionat? *

Da

Nu

Indicați nivelul de pregătire pe care îl aveți în depistarea și scrierea unui raport/ fișe de consulație pentru o suspiciune de abuz: *

	1	2	3	4	5	
Absent	○	○	○	○	○	Înalt

Care este motivul pentru care ați raporta, un caz de abuz autorităților? *

- Pentru a proteja animalul
- Pentru a proteja persoanele din familia respectivă
- Principii etice
- Codul de conduită profesională
- Politica angajatorului
- Din cauze legale

Mod de previzualizare

Publicat

Copiați linkul respondentului

Acest formular nu acceptă răspunsuri.

Gestionează setările de publicare

Da

- Nu
- Altele:

Cât de familiarizat sunteți cu noțiunea de abuz prin neglijență? *

	1	2	3	4	5	
Deloc	○	○	○	○	○	Stăpânesc domeniul foarte bine

Ce ați include în categoria abuzului prin neglijență? *

- Cahexia
- Obezitatea
- Bolile cronice netratate
- Boli acute/dureroase lăsate fără asistență medicală
- Colecționarea de animale de companie
- Altele:

Mod de previzualizare

Publicat

Copiați linkul respondentului

Acest formular nu acceptă răspunsuri.

Gestionează setările de publicare

- Da
- Nu îmi dau seama
- Nu

Considerați că aveți nevoie de pregătire suplimentară în acest domeniu, pentru identificare și raportarea unui abuz?

- Da
- Nu
- Altele:

În general, cum ați descrie relația pe care o aveți cu proprietarii? *

- În general ii privesc ca pe niște parteneri, cu care colaborez
- Tind să fiu mai autoritar, să îmi impun punctul de vedere, fără să iau în calcul opțiunile lor
- De cele mai multe ori tind să ma las condus de părerile și planurile lor

Mod de previzualizare

Publicat

Copiați linkul respondentului

Acest formular nu acceptă răspunsuri.

Gestionează setările de publicare

Da

Nu știu

Nu

Aici puteți să împărtășiți cu noi orice gând legat de subiectul abordat. Vă mulțumim!

Răspunsul tău

Trimite

Golește formularul

Nu trimiteți parole prin formularele Google.

Acest conținut nu este nici creat, nici aprobat de Google. - Contactează proprietarul formularului - Condiții de utilizare - Politica de confidențialitate

Acest formular pare suspect? Raportează

Formulare Google

Mod de previzualizare

Publicat

Copiați linkul respondentului

Acest formular nu acceptă răspunsuri.

Gestionează setările de publicare
