# Supplementary material for: Animal Abuse and Neglect in Companion-Animal Practice: The Role of Training, Legislation, and Veterinarian–Client Relationships in Romania
Source: Vet Sci. 2026 Jul 17;13(7):696. doi: 10.3390/vetsci13070696 (PMC13418691; doi:10.3390/vetsci13070696)
Supplement: Supplementary file 1 [file vetsci-13-00696-s001.zip › Supplementary Material S2 English Translation of the Questionnaire Used in the Study.pdf]

Questionnaire for Veterinarians –
Analysis of the Management of Abuse Cases against Owned Companion Animals

This questionnaire is addressed to veterinarians with the right to practice independently on the territory of Romania. Its purpose is to collect data necessary for a study analyzing how cases of abuse, including abuse by neglect, against owned companion animals are managed.

Please consider your own experiences encountered in practice. We do not collect or use personal data.

The data provided will be stored and analyzed on the Google Drive platform, which has taken the necessary measures to comply with the requirements of Regulation (EU) 2016/679 on the protection of natural persons with regard to the processing of personal data and on the free movement of such data, and repealing Directive 95/46/EC (General Data Protection Regulation).

The research team is mixed, comprising a supervising professor, a veterinarian, and a psychologist, PhD candidate at the Faculty of Veterinary Medicine, USAMV Bucharest. The data obtained will be published in the form of a doctoral thesis.

* Indicates a required question

---

1. You carry out your veterinary activity within a: *

- Companion animal veterinary practice/clinic
- Mixed veterinary practice/clinic (companion animals and farm/production animals)
- Veterinary hospital dedicated to companion animals
- Other:

2. Age: *

- 24–30 years
- 30–40 years
- 40–50 years
- 50–60 years
- Over 60 years

3. You practice in a: *

- Urban environment
- Rural environment
- Both

4. In which county do you practice? *

[Dropdown selection]

5. Years of experience: *

- 1–5 years
- 6–10 years
- 11–20 years
- 21–30 years
- Over 30 years

6. You identify as: *

- Female
- Male

- Non-binary
- Other

7. How familiar are you with the legislation regulating animal abuse? *

Scale 1–5   1 = Not at all familiar with this topic | 5 = Very familiar

8. Have you received training on identifying and managing abuse cases? *

- Yes
- No

9. Do you have (at your workplace) procedures to be applied in cases of suspected abuse? *

- Yes
- No

10. Do you know which institution you should report suspected abuse cases to? *

- Yes
- No

11. Have you encountered cases in which you suspected animal abuse? *

- Yes
- No

12. In how many cases have you suspected animal abuse in the last 12 months? *

- No cases
- Between 1–5 cases
- 5–10 cases
- 10–20 cases
- Over 20 cases

13. What do you think is the most frequent form of animal abuse? *

- Physical abuse (resulting from physical violence, beatings, use of sharp weapons, hanging/strangulation, burns, poisoning, etc.)
- Sexual abuse
- Abuse by neglect
- Abuse through organizing animal fights
- Abandonment and/or driving away of animals
- Separating mothers from offspring before they reach 8 weeks of age
- Failure to ensure welfare conditions (inadequate shelter, short chain <2 m, small enclosure, inadequate food and water, etc.)
- Other:

14. Have you ever reported a case of abuse to the authorities? *

- Yes
- No

15. Have you ever completed a report/consultation form/official document regarding a situation of abuse, clearly describing the suspected abuse? *

- Yes
- No

16. Indicate your level of preparedness in detecting and writing a report/consultation form for a suspected abuse case: *

Scale 1–5   1 = Absent | 5 = High

17. What is the reason for which you would report a case of abuse to the authorities? *

(multiple selection)

- To protect the animal
- To protect the family members involved
- Ethical principles
- Professional code of conduct
- Employer's policy
- Legal reasons

18. If you suspected abuse and intended to report it, would you also inform the owner? *

- Yes
- No
- Other:

19. How familiar are you with the concept of abuse by neglect? *

Scale 1–5   1 = Not at all | 5 = I master the subject very well

20. What would you include in the category of abuse by neglect? *

- Cachexia
- Obesity
- Untreated chronic diseases
- Acute/painful conditions left without medical care
- Companion animal hoarding
- Other:

21. Have you found yourself in a situation where you provided the owner with information, or educated them, regarding aspects that may fall under the category of abuse by neglect (obesity, malnutrition, untreated chronic diseases)? *

- Yes
- I don't know
- No

22. Do you consider that you need additional training in this field, for identifying and reporting abuse?

- Yes
- No
- Other:

23. In general, how would you describe the relationship you have with owners? *

- I generally view them as partners I collaborate with
- I tend to be more authoritative, imposing my point of view without considering their options
- Most often I tend to let myself be guided by their opinions and plans

24. At the end of the consultation, does the owner receive a copy of the consultation form, in physical or electronic format? *

- Yes
- I don't know
- No

25. Here you can share with us any thoughts related to the topic discussed. Thank you!

[Open text response field]
