# Supplementary material for: Animal Abuse and Neglect in Companion-Animal Practice: The Role of Training, Legislation, and Veterinarian–Client Relationships in Romania
Source: Vet Sci. 2026 Jul 17;13(7):696. doi: 10.3390/vetsci13070696 (PMC13418691; doi:10.3390/vetsci13070696)
Supplement: Supplementary file 1 [file vetsci-13-00696-s001.zip › Supplementary Material S3 Ethics Committee Approval.pdf]

UNIVERSITATEA DE ȘTIINȚE AGRONOMICE
ȘI MEDICINĂ VETERINARĂ – BUCUREȘTI

UNIVERSITATEA DE ȘTIINȚE AGRONOMICE
ȘI MEDICINĂ VETERINARĂ - BUCUREȘTI
IEȘIRE Nr. 2625
Ziua 18 Luna 06 Anul 2026

CEU 4/18.06.2026

Comisia de Etica a USAMV Bucuresti avizează favorabil solicitarea de examinare a cererii referitoare la conformitatea articolului "***Animal abuse and neglect in companion-animal practice: the role of training, legislation, and veterinarian – client relationships in Romania***" cu autorii Adela Ioana Mustățea, Violeta Stefania Rotărescu, Adrian Gorbănescu și Emilia Ciobotaru-Pîrvu (cadre didactice și doctoranzi ai USAMVB și ai Universității din București) ce va fi trimisă spre publicare la revista **Veterinary Sciences (MDPI).**

Se certifică faptul că acest articol respecta normele și reglementările etice din Universitatea noastră, ce derivă din Regulamentul (UE) 2016/679 și Legea nr. 190/2018 aplicabile în Romania. Certificăm că studiul a fost realizat în conformitate cu liniile directoare ale Declarației de la Helsinki.

In concluzie, articolul este conform din punct de vedere etic, iar utilizarea datelor și a materialului biologic este justificată, transparentă și realizată cu respectarea drepturilor și intereselor tuturor părților implicate.

Data: 17.06.2026

Comisia de etică universitară - Subcomisia de etica cercetării științifice din USAMV București

Prof.univ.dr. Călina Petruța Cornea

Prof.univ.dr Minodora Tudorache

Student Serena Bucur

WWW.USAMV.RO
B-DUL MĂRĂȘTI 59, 011464 – BUCUREȘTI
TEL. CENTRALĂ: +4 021 318 2564 ■ TEL. RECTORAT: +4 021 318 2266 ■ FAX: +4 021 318 2888 ■ E-MAIL: POST@INFO.USAMV.RO
